# The Epistemic Limits of Impactful Dreams: Metacognition, Metaphoricity, and Sublime Feeling

**DOI:** 10.3390/brainsci14060528

**Published:** 2024-05-22

**Authors:** Don Kuiken

**Affiliations:** Department of Psychology, University of Alberta, P217 Biological Sciences Building, Edmonton, AB T6G 2E9, Canada; dkuiken@ualberta.ca

**Keywords:** metacognition, metaphor, sublime feeling, impactful dreams

## Abstract

Taxonomic studies of dreams that continue to influence the dreamer’s thoughts and feelings after awakening have distinguished three types of impactful dreams: nightmares, existential dreams, and transcendent dreams. Of these, existential dreams and transcendent dreams are characterized by recurrent metacognitive appraisal of the epistemic tension between complementary (a) metaphoric (A “is” B) assertions and (b) literal (A “is not” B) assertions. Metacognitive appraisal of such complementary metaphoric and literal assertions is detectable as the felt sense of inexpressible realizations. The poesy of such inexpressible realizations depends upon the juxtaposition of a metaphoric topic and vehicle that are both “semantically dense” but at an abstract level “distant” from each other. The result is “emergence” of attributes of the metaphoric vehicle that are sufficiently abstract to be attributes also of the metaphoric topic. The cumulative effect of successive metaphoric/literal categorical transformations produces a higher-level form of metacognition that is consistent with a neo-Kantian account of sublime feeling. Sublime feeling occurs as either sublime disquietude (existential dreams) or as sublime enthrallment (transcendent dreams). The aftereffects of these two dream types are thematically iterative “living metaphors” that have abstract (but not “totalizing”) ontological import.

## 1. Introduction

Following spontaneously recalled dreams, dreamers sometimes sense immediately what the dream “means”, and the dream’s “meaning” continues to influence the dreamer’s thoughts and feelings for some time afterwards. In seminal studies of these exceptional oneiric events, Jung [1] (p. 117) referred to “big” dreams; Hartmann [2] (p. 54) focused on “intense” dreams; and States [3] (p. 238) described dreams with “magnitude”. The lingering effects of these exceptional dreams may depend upon the kind of semantic complexity that literary scholars attribute to metaphoric (poetic) expression [4]. By implication, oneiric access to semantic complexity may depend upon the same mode of metacognition that initiates waking metaphoric (poetic) expression. While dreaming in general (regardless of sleep stage) lacks the metacognitive nuances of waking cognition [5], impactful dreams may nonetheless involve the same modes of metacognition that support waking aesthetic experience.

In some instances, dream impact seems to derive from the dream per se: the manifest dream presents compelling meanings that defy literal paraphrase. However, the effects of impactful dreams often exceed their manifest content and involve (a) their state-transitional endings (immediate carryover effects) and (b) their lingering aftereffects (response to dream reminders). The interdependent components of this temporal sequence may comprise a *gestalt* [6] that arises from epistemic tension between the metaphoric “is” and literal “is not” of a sequence of semantically resonant dream images [7,8]. Through a process that extends from the manifest dream to the dream ending and waking reminders, the effects of this epistemic tension plausibly include representation of compelling abstract-ontological (“totalizing”) categories.

### 1.1. Impactful Dream Types: Manifest Content

It is difficult to determine whether these exceptional (hereafter “impactful”) dreams are of different types (“species” [9]) or whether they occupy extreme points on a selected continuum (e.g., “dream-like fantasy” [10]; “immersion”, [11]). Although there are taxometric procedures for systematically differentiating continuous from typological constructs [12], researchers studying impactful dreams have usually not availed themselves of that psychometric technology. However, using an established approach to typological clarity [13], a series of taxonomic studies has revealed three types of impactful dreams, each distinguished by features involving feelings and emotions, narrative themes, movement characteristics, sensory anomalies, spontaneous transformations, effectuality/ineffectuality, and concluding affective tone (see Table 1).

#### 1.1.1. Primary Emotions

The results of these studies suggest a tripartite classification based on three primary emotions, including affectively congruent narrative themes (fear/anxiety with harm avoidance, sadness/despair with response to loss, ecstasy/awe with magical accomplishment). This pattern makes it tempting to distinguish impactful dreams simply according to their predominant emotional tone and affectively congruent narrative themes. However, an attempt to differentiate impactful dreams in this way would elide other distinctive features of these dream types. Potentially elided features include the spontaneous transformations that characterize all three impactful dream types and the capacity for dual perspectives that is specific to existential and transcendent dreams.

#### 1.1.2. Spontaneous Transformations

Unlike mundane dreams, impactful dreams include transformations of dream content (physical metamorphoses in nightmares, spontaneous feeling shifts in existential dreams, and spontaneous perspective shifts in transcendent dreams). It is conceivable that these transformations are merely part of the imaginal “drift” that follows REM sleep decoupling of the dorsal attentional network from the anterior portions of the default network [19]. However, while the default network model addresses the decline in attention to the external world that separates dreaming from waking cognition and explains the imaginal simulation of a seemingly “real” world, it does not address the role of attentional adjustments in the transformations that characterize REM sleep. Contemporary neurophysiological models have tried to address these attentional adjustments.

The activation synthesis model [20] originally suggested a single oneiric attentional adjustment. Ponto-geniculo-occipital (PGO) activation during REM sleep purportedly generates diffuse activation of the dorsal attentional network without regulation by forebrain executive networks. The result of this disjunction is chaotic (“bizarre”) dream narratives. The AIM extension of this model [21] retained focus on a single attentional adjustment but did allow that PGO waves during REM sleep do not simply produce radically chaotic dream narratives. Instead, PGO activation triggers discontinuities, and mnemonic elements following a discontinuity were transformations of a prior mnemonic element (e.g., a car becomes a bike). Rittenhouse et al. [22]; see also [23] began to document these transformations, concluding that, “despite the threat to coherence posed by bizarre discontinuities, significant coherence is maintained by associational constraints” (p. 100).

In his contemporaneous neurophysiological model, Morrison [24] also portrayed PGO activation during REM sleep as the phasic marker of a single attentional system: “the startle network”. However, he subsequently refined this account to address the “critical behavioral response of orienting, with startle being a less organized expression of a response to an unexpected stimulus” [25] (p. 171). Although these two forms of “alertness” (startle and orienting) are amply documented in the attention literature [26,27], the oneiric transformations they generate during REM sleep have not been clearly differentiated [28].

Differentiating these types of transformation may require consideration of contrasting patterns of activity within the autonomic nervous system (ANS). Recent evidence confirms that, in response to loud tones, post-traumatic and idiopathic nightmares are associated with susceptibility to startle and increased skin conductance, while idiopathic nightmares are associated specifically with increased skin conductance [29]. This pattern suggests that increased sympathetic activation—with decreased parasympathetic activation—is the ANS substrate of susceptibility to attentional startle and full-bodied arousal during nightmares [30]. In contrast, increased parasympathetic activation—with decreased sympathetic activation—may be the ANS substrate of the heart rate deceleration and postural-kinaesthetic inhibition that precedes attentional reorienting. Such neuromuscular inhibition is the distinctive “pause” that precedes the orienting response—and that distinctively marks existential and transcendent dreams.

Kuiken and colleagues [17] articulated such a dual process model, although empirical support remains limited to studies that rely on self-report rather than neurophysiological evidence of ANS-modulated attentional adjustments. First, the balance of sympathetic and parasympathetic ANS activation plausibly influences the sensory modalities that predominate in the transformations within each dream type. The physical metamorphoses of nightmares involve transformations in the distal (primarily visuospatial) sensory modalities that are salient during the hyperarousal of post-traumatic and perhaps idiopathic nightmares [31]. In contrast, the spontaneous transformations of existential and transcendent dreams involve proximal sensory modalities (affective and kinaesthetic modalities in existential dreams; postural and vestibular modalities in transcendent dreams).

Second, the balance of sympathetic and parasympathetic ANS activation plausibly influences the kind of transformation that is characteristic of each dream type. Sympathetic ANS activation may support the physical metamorphoses of nightmares. These transformations are ampliative, i.e., progressively startling, alarming, or even catastrophic. For example, in one prototypic nightmare [14], a dreamer who was attacked by a metal dog reported panic when the dog came to life and “that’s when I woke up very scared”. In contrast, parasympathetic ANS activation supports the quasi-metaphoric transformations of existential and transcendent dreams. These transformations involve categorial reorienting, as though the dream is implicitly addressing the question, “What is this?” And, the implicit answer is, “Not X, but a categorial transformation X’”. If the orienting response is intense, a relatively remote conceptual neighbor may be activated. For example, thoughts of “aphids on the leaves of my tomatoes” may be transformed into a semantically resonant image of “spiders on my hands” [32].

#### 1.1.3. Dual Perspectives

A distinctive form of metacognition may give the spontaneous transformations of existential and transcendent dreams their epistemic import. There is replicable evidence [14,15,17,18] that existential dreams and transcendent dreams involve a form of reflective awareness in which dreamers sense themselves from two points of view (e.g., “I became split into two parts; I was able to experience the dream world from either perspective”). Such dual attunement not only departs from the single-mindedness of typical dreams [33] but also from the concurrent awareness of dreaming that is the allure of lucid dreaming [34]. The proposal offered here is that the dual attunement evident in existential and transcendent dreams enables metacognitive attunement to the metaphoricity of existential and transcendent dreams.

Metacognition during REM and N2 sleep occurs much less frequently than during wakefulness [5]. Nonetheless, oneiric metacognition is strikingly evident when dreamers become aware of dreaming while dreaming [35] and routinely evident when dreamers reflect on their ongoing thoughts, feelings, and behavior [18,35]. In contrast to these self-reflective moments, the distinctive form of metacognition in existential and transcendent dreams involves an epistemic tension that Searle [8] argued is a fundamental aspect of metaphor comprehension (see also [36]). He proposed that metaphor comprehension involves dual attunement to (a) a *metaphoric* (A “is” B) assertion and (b) a complementary *literal* (A “is not” B) assertion. For example, to say that my surgeon is a butcher brings attention to the metaphoric truth value of a statement (e.g., my surgeon [metaphorically] “is” a butcher) and simultaneously to its literal falsehood (e.g., my surgeon [literally] “is not” a butcher).

The conflict between these assertions induces a reflectively accessible (“felt”) tension [7] that has epistemic import. The present proposal is that metacognitive attunement to (a) a metaphoric (A “is” B) assertion and (b) a complementary literal (A “is not” B) assertion contributes to the impact of existential and transcendent dreams.

#### 1.1.4. Manifest Content: Implications

The manifest content of taxonomically delimited impactful dreams affirms the complexity required to explain their influence on waking thoughts and feelings. Emphasis on spontaneous transformations, rather than on the dream’s dominant emotional tone, motivates examination of how a metacognitive tension during existential and transcendent dreams supports their distinctive metaphoricity. This proposal contrasts with formulations that acknowledge continuity between cognition during wakefulness and during REM dreaming [19]—and restrict dream metaphoricity to retrospective reflection [37,38]. The distinctive form of metacognition in existential and transcendent dreams underscores the lively metaphoricity of dream cognition per se.

## 2. Intrinsic Oneiric Metaphoricity

### 2.1. Spreading Activation and the Metaphoricity of Dream Cognition

To clarify the metaphoricity of impactful dreams, it is useful to consider first the “creativity” of typical REM dreams. Theories of creativity often emphasize problem solutions that result from the generation of remote associations between existing concepts. That basic account has generated discussions of oneiric creativity that emphasize the increased accessibility of associatively remote lexical items during REM sleep [23] and temporally remote memories during late night sleep [39]. Available research indicates that (a) normatively weak lexical associations, ranging from hierarchical semantic relations to mere contiguity, are strengthened following REM sleep [23]; (b) normatively weak associations to emotion words are strengthened when they are primed by presentation of those emotion words prior to REM sleep [40]; and (c) an “association” that captures the (schematic) gist of an array of semantically related words, ranging from part-whole to contextual relations, is “remembered” following REM sleep [41,42]. These results have been presented as evidence that memory traces activate relatively distant, uncommon, and novel semantic associations during REM sleep.

It has been tempting (cf. [43]) to generalize this spreading activation model to explain higher order “creative” phenomena. However, such generalization is problematic partly because, in the studies just mentioned, the weak associations strengthened during REM sleep range from those based on mere contiguity (word co-occurrence) to those involving basic semantic relations (e.g., noun-noun relations, adjective-noun relations). Generalization of the spreading activation model would be more compelling if the weak associations under investigation differentially and systematically included (a) categorial taxonomic relations (e.g., hypernyms, hyponyms, class co-membership); (b) part-whole relations (e.g., characteristic features, traits); (c) thematic relations (e.g., characteristic possessions, locations); and (d) figurative modifier-modified relations [44]. Generalization of the spreading activation model would be even more compelling if the weak associations under investigation included not only the figurative modifier-modified relations of mundane non-literary metaphors (e.g., “genes are blueprints”) but also the figurative modifier-modified relations of unconventional poetic metaphors (e.g., “death is a fat fly”).

### 2.2. Carryover Effects and Mundane Dream Metaphoricity

To clarify the poetic metaphoricity of impactful dreams, it is useful to begin by considering the mundane metaphoricity of typical REM dreams. It is well established that awakenings from REM and NREM sleep are followed by a brief period (20–40 min) of changes in affect, perception, cognition, and memory (“carryover effects”). The carryover effects of typical REM dreaming include readiness to identify rudimentary figurative relations. Cai et al. [45] provided evidence that REM sleep reactivation of analogical relations (e.g., “chips” are related to “salty” as “candy” is related to “sweet”) influenced post-awakening performance on a task requiring identification of word combinations that included a root term from the analogy (e.g., sweetheart, sweet sixteen, sweet cookies). This task involves identification of modifier-modified compounds that can be paraphrased as conventional nominal metaphors (e.g., in your heart you are sweet, being sixteen is sweet).

However, the relevance of this version of the remote associates task (RAT) for assessing metaphoricity is obscured by the additional challenge of identifying three compounds involving the same root term. This additional interpretive complexity may make the RAT a useful measure of a certain kind of creative problem solving [46,47,48], especially the inference-driven interpretation that is characteristic of the evaluative component of creative cognition (e.g., recognizing relations among response alternatives, reaching single solutions to complex problems). However, the Cai et al. [45] results do not reflect the carryover of metaphoricity per se.

### 2.3. Carryover Effects and Impactful Dream Metaphoricity

There is accumulating evidence [49,50] that, rather than separately or additively, the interactive combination of generative and evaluative processes predicts creativity. For example, generating as many different associations as possible to target words (associative fluency) predicts creativity [51,52]; providing as many associations as possible that are unrelated to target words (associative restraint) also predicts creativity [52]. However, the carryover effects of existential and possibly transcendent dreams (but not nightmares) include neither associative fluency nor associative restraint separately but rather their interactive combination [53]. Moreover, in the latter studies, the interactive combination of associative fluency and associative restraint was unrelated to performance on the RAT that Cai et al. [45] used to assess the metaphoricity of typical REM dreaming. By implication, the metaphoricity of existential and transcendent dreams does not involve recognizing relations among response alternatives and reaching single solutions to complex problems as measured by the RAT. Instead, the metaphoricity of impactful dreams may involve a more nuanced interplay of associative activation and associative restraint, perhaps sufficiently nuanced to explain the “involuntary poetry” of impactful dreams [3].

### 2.4. Metacognitive (Noetic) Feelings

Metaphor generation [54,55], lyrical improvisation [56], and musical improvisation [57,58] involve rapidly oscillating—or even simultaneous—activation of generative and evaluative neural networks [59]. Moreover, tightly woven interplay between generative and evaluative neural networks may prompt “automatic-affective appraisals” [50]. Reference to automatic-affective appraisals invites closer consideration of the tension between the metaphoric (generative) “is” and literal (evaluative) “is not” of oneiric metaphoricity. Metacognitive appraisal of this metaphoric/literal tension is one type of noetic (epistemic) feeling; others include (a) feelings of knowing [60]; (b) tip-of-the-tongue feelings [61]; (c) feelings of familiarity [62]; and (d) feelings of ‘déjà vu’ [63]. Metacognitive appraisal of metaphoric/literal tension can, with some care and caution, be distinguished from feelings toward independently perceived or imagined intentional objects, such as (a) external states of affairs (e.g., “I feel fond of X”) or (b) bodily states and dispositions (e.g., “I feel tired”). However, metacognitive appraisal of the tension between a metaphoric “is” and literal “is not” is also experienced differently—and functions differently—during dreaming than during waking.

#### 2.4.1. A Metacognitive “Felt Sense” of Metaphoric/Literal Tension

Feelings and emotions in existential and transcendent dreams involve proximal sensory modalities that “soften” the way they are “given” in experience. The affective-kinaesthetic modalities of existential dreams and the postural-vestibular modalities of transcendent dreams give dream feelings sensuous palpability (e.g., sadness that involves sensed weakness or inability to move, joy that involves sensed floating or flying). Metacognitive metaphoric/literal tension during dreaming may similarly occur as a softly felt affective moment, a “felt sense” [64] of tension between a metaphoric “is” and literal “is not”.

#### 2.4.2. Attentional Reorienting, Category Transformation, and Metaphoric/Literal Tension

Although attention network theory [27] distinguishes three independent systems (vigilant anticipation of selected stimuli; reorienting adjustment to unanticipated stimuli; controlled selection from available stimuli), the attentional network that distinctively shapes existential and transcendent dreams is the orienting response system. On one hand, recent evidence indicates that, during creative response to constrained abstract problems (e.g., multiple uses tasks, interpreting analogies), sustained selective attention is complemented by an executive function (updating working memory) that facilitates access to “different problem solutions” [65]. On the other hand, during a creative response to extended imaginative problems (e.g., metaphor comprehension), sustained selective attention is complemented by an executive function (shifting between mental sets) that facilitates perspective shifts to “different dimensions” [65] and “levels of analysis” [66].

Perspectival shifts to different dimensions and levels of analysis of the same (or a similar) categorial object have been observed within the oddball paradigm that is commonly used to examine the orienting response during waking [65,66]. During dreaming, the primary function of oneiric metacognitive appraisal may be to monitor relations between the successive categorical representations that spontaneously occur during PGO generation and ANS modulation of the perspectival shifts triggered by the orienting response. These shifts involve categorial reorienting, experienced as the transition from one representation of the category (e.g., aphids on my tomatoes) to semantically resonant representation of the same or a similar category (e.g., spiders on my hands).

#### 2.4.3. Openness to Experience and Metaphoric/Literal Tension

The contrast between (a) attentional openness to alternative solutions for constrained abstract problems and (b) attentional openness to different levels or dimensions of sensuous-affective categories is congruent with the distinction between creative instrumental and creative experiential sets [67]. For some creative individuals, shifts in attention occur as part of an instrumental exploration of different problem solutions. For others, shifts in attention occur as part of an experiential transition in which attention is reoriented toward different dimensions or levels of sensuous-affective categorial representations. Different facets of trait openness to experience (Intellect, Openness) reflect this contrast [68,69]; moreover, trait absorption (Tellegen Absorption Scale; TAS) [70] is most closely related to the Openness facet [71]. Understandably, then, the TAS predicts the inexpressible realizations that emerge from existential dreams [72]—although that relationship is not evident for transcendent dreams.

#### 2.4.4. Performative Improvisation and Metacognitive Appraisal

Metacognitive appraisal of metaphoric sameness and literal difference may involve attunement to transitions from one sensible aesthetic representation to semantically resonant aesthetic representations during improvisatory performance [73,74,75,76]. The performative aspect of metacognitive attunement to extended metaphor is arguably the generative “life” of extended metaphors that Ricoeur [7] calls “living metaphor”. Such metacognitive attunement may also be the generative core of literary authorship. Poets—and some dreamers—often reshape dream imagery to “carry forward” intimations of its affective tone and categorial nuance long after awakening from an impactful dream [3,77]. Rather than veridical and detailed description of a remembered dream, imagery from the manifest dream subtly foreshadows expressive departures from mundane reflection, routine remembering, and everyday reasoning. The dream prefigures and the poet extends the dream’s meaning by elaborating its figurative configurations beyond what can, at best, be partly captured by a carefully framed literal paraphrase.

## 3. Temporally Extended Metaphoric Interplay

Continued metacognitive appraisal of the tension between metaphoric and complementary literal assertions supports the integration of successive metaphoric/literal representations. While it is possible to identify metaphoric categorial representations analytically, it is especially important to articulate their progressive integration. To clarify this objective, what follows is a linguistic and then an oneiric example of such temporally extended metaphoricity.

### 3.1. A Generic Linguistic Example

The loci of such progressions and interplay are usually identifiable, as in the following excerpt from Borges’ [78] essay, “A New Refutation of Time”. This excerpt is useful here because it is neither a literary text nor a dream report. In these lines, an understanding of time is metaphorically (and progressively) represented during reflective consideration of its moving, devouring, and immolating insistence:

Time is a river that carries me along,
and I am the river;
It is a tiger that devours me,
and I am the tiger;
It is a fire that consumes me,
and I am the fire.

In this excerpt, several local figurative features stand out. First, each line begins with a simple nominal metaphor (e.g., “Time is a river”). Second, each nominal metaphor is followed by elaborative (i.e., explicative, perhaps even ampliative) modulation of an initial metaphoric vehicle (e.g., “… it [the river] carries me along”). Third, each nominal metaphor with its elaborative modulation is followed by compound metaphoric modulation involving a nominal first-person metaphor (e.g., “I am the river”) that has both the initial metaphoric vehicle (e.g., “a river”) and, indirectly, the initial metaphoric topic (e.g., “time”) as its metaphoric topics (e.g., “I am” river-like time).

And yet, beyond these local figurative features, a higher order figurative structure is also evident. Although Borges originally wrote this passage as prose, its three-part “verse” structure (accentuated by the line breaks added here) supports (a) three separate first-person compound metaphoric modulations (“I am” [river-like] time; “I am” [tiger-like] time; “I am” [fire-like] time) and (b) an integrative first-person compound metaphoric modulation (“I am” [river-like, tiger-like, fire-like] time). Are the separate (iterative) and inclusive (integrative) effects of these metaphoric modulations independently evident to actual readers of these lines? Some empirical evidence indicates that they are. In an empirical study of young adult readers, Kuiken and Douglas [79] (Study 1) found that the separate (iterative) first-person compound forms (“I am [river-like] time”; “I am [tiger-like] time”; and “I am [fire-like] time”) prompted reports of inexpressible realizations (e.g., “I sensed something that I could not find a way to express”). They also found that the inclusive (integrative) compound form (“I am” [river-like, tiger-like, fire-like] time) distinctively precipitated inexpressible realizations with a concomitant sense of release. The latter result is consistent with informal observations [64] that such an ineffable realization is a “felt shift” accompanied by a momentary “sense of release” as though there is a subtle “letting go”, a subdued and softened “Oh, that’s there”. This metacognitive appraisal marks an apex moment of sublime feeling.

### 3.2. An Oneiric Example

Comparably compounded metaphoric modulations often occur in impactful dreams. In the following prototypic existential dream (from [80], p. 136), a three-episode structure (analogous to the Borges three-verse structure) progressively—and metaphorically—discloses the dreamer’s felt sense of the coercive character of inauthentically intimate relationships.

***The Hotel Dream.*** [I was in] a hotel in southern Alberta or someplace. I was traveling by myself, I think, and I remember worrying about rapists in the hall and this sort of thing. I remember thinking, “Well, I’ll have to be brave because I’m by myself”. So I took this room in a secluded area of the hotel … and anyway it seemed to work out. // And then this hotel seemed to be in France. My family was with me, and we were all in a room together. We were packing to leave. I was very organized; I had all of my stuff ready to go … My family was very disorganized and I was having to help them. I didn’t want to. I thought, “Well, they can do it themselves; I’m not responsible for their packing”. But it was almost impossible not to help them because I needed another bag or two and I had things stored in a particular drawer and they had dumped all their stuff in there, too. So in order for me to get this packing done, I had to help them anyway // [Then I think I had gone off on my own for a while] and I came back [to the hotel again.] I got a phone call, an overseas phone call from my Dad … He had gotten a doctor’s report, and the doctor said that he [had an ailment that] would never heal. And I had plans about my whole family moving to France … but he just told me how sick he was and that he would never heal. And there was some stupid person on the phone … some practical sounding person, who was sticking her nose in there. I kept telling her to shut up … and I was really upset and crying very hard. My Dad said that he wanted to talk to my Mom. So my Mom came to the phone [and she thought that it wasn’t practical to live in France]. She seemed to think that it was better to stay in Canada. I was surprised by her ability to say what was best for me … and I remember trying to talk her into it. I was overwhelmed by the fact that my father would never heal. I couldn’t be with him and also stay in France. I woke up crying. I was just really sad. I felt this sadness just coming out of the bottom of my soul, from way down deep some place.

In this dream, three successive episodes (separated here by //) present semantically resonant portrayals of concern about coercive intimacy (worry about rapists in an Alberta hotel hallway; unwelcome assistance to filial fellow travelers in a hotel in France; aversion to a series of familiar voices who seem to know what is “best” for the dreamer). Although less simply than in the Borges lines that present compound metaphoric representations of “time”, this three-episode structure presents compound metaphoric representations of the coercive character of inauthentically intimate relationships.

Given persistent self-representation in dream narratives, this dream presents a series of three separate first-person compound metaphoric modulations (“I am here” [metaphorically] living with the possibility of rapists in the hallway; “I am here” [metaphorically] living in opposition to de facto filial collaboration; “I am here” [metaphorically] living with dispersed (parental) presumptions about what is best for me). As in the Borges lines, the result is a repetitive structure (a recurrent theme) that involves three separate first-person compound metaphoric modulations. Beyond that, an integrative higher order first-person compound metaphoric modulation is established by the series of first-person metaphoric modulations moving from (a) “I am here” [metaphorically] living with the possibility of rapists in the hallway to (b) “I am here” [metaphorically] living in opposition to de facto filial collaboration and then to (c) “I am here” [metaphorically] living with dispersed (parental) presumptions about what is best for me. This sequence is more clearly ampliative than the sequence of Borges lines, moving the dreamer toward a dramatic and mournful sense of release: She concludes, “I felt this sadness just coming out of the bottom of my soul, from way down deep some place”. This second level of metacognitive metaphoric/literal appraisal marks an apex moment of sublime feeling.

More complete articulation of the unfolding metaphoric structure of this dream would require the scrutiny derived from additional (open-ended) questions about nuances in the dream narrative [81]. Perhaps, however, the preceding analysis is sufficient to clarify how the entirety of this dream is analogous to the entirety of the highly structured Borges lines in the way it moves toward first-person metaphoric articulation of a theme and toward a poignant moment of sublime feeling—all within the dream and prior to waking reflection.

### 3.3. A Hierarchy of Figurative Relations

The preceding account relies upon a hierarchy of figurative relations: (a) detectable image resonance; (b) detectable unidirectional metaphoricity; (c) detectable bidirectional metaphoricity; and (d) metaphors of personal identification. The first level of metacognition moves toward detectable bidirectional metaphoricity and emergent meanings; the second level of metacognition moves toward metaphors of personal identification and sublime feeling.

#### 3.3.1. Detectable Semantic Resonance

In dream reports [82,83], narrative discontinuities often juxtapose neighboring instances of the same categorial concept (e.g., a car and a bike as means of transportation [22]; a person and a statue as human forms [84]). During impactful dreams, the dreamer may “sense” semantic resonance between successive neighboring representations—even without the syntactic structure that might, in another context, link them as the topic and vehicle of nominal metaphors (as occurs with modifying-modifier compounds such as “statue people”, “flute birds” [85]). The possibility of such accentuated—and detectable—resonance is reinforced by evidence that awakening from existential dreams, transcendent dreams, and nightmares is followed by increased readiness to include metaphoric vehicles and topics in the same conceptual category (e.g., “genes” and “blueprints” taken from the nominal metaphoric assertion “genes are blueprints”). Post-dream detection of such semantic resonance is specific to vehicles and topics taken from *conventional* literary and *conventional* non-literary metaphors [53] (Study 2). Individuals participating in dream research may sense this kind of semantic resonance as indication that their dream was “impactful”, i.e., that it affected—and continues to affect—their thoughts and feelings after awakening.

#### 3.3.2. Detectable Unidirectional Metaphoricity

In empirical studies of dreaming, conceptual metaphor theory has often been used to explain how a metaphoric vehicle is unidirectionally “mapped” onto a metaphoric topic [86]. According to a summary provided by Malinowski and Horton [38] (p. 10), (a) dreams can concretely picture something abstract from waking life; (b) dreams can be about emotional aspects of the dreamer’s life; and (c) it may be necessary to talk with the dreamer about current life events to understand the metaphor. In general, the function of unidirectional metaphoric reference to waking life is to generate novel self-understanding during wakefulness [37].

The nature of unidirectional mapping must be considered carefully; two aspects of this process entail interaction between the vehicle and topic prior to such mapping. First, the determination of which vehicle attributes are mapped onto the topic depends upon the selection of salient attributes of the vehicle that were not salient attributes of the topic prior to unidirectional mapping [87]. Second, vehicle attributes may be transformed prior to unidirectional mapping onto the topic. There is no consensus about how such attribute transformations are derived, although likely sources include polysemy, analogy, and extended metaphor [88]. These forms of interplay between vehicle and topic determine the nature of unidirectional mapping—but disclose nothing novel about the vehicle.

#### 3.3.3. Detectable Bidirectional Metaphoricity

In contrast, the present framework focuses on the disclosive potential of bidirectional metaphoric structures in existential and transcendent dreams. A primary source of this potential is the distinctive bidirectionality (A “is” B and B “is” A) of poetic metaphors. While Lakoff and Turner [89] dismissed bidirectional mapping, a tradition extending from Richards [90] and Black [91] to Fauconnier and Turner [92] and Goodblatt and Glicksohn [6] provides articulation of the creative potential of bidirectional metaphor comprehension. In contrast to unidirectional mappings (A “is” B; “death is a fat fly”), the bidirectional epistemic import of the vehicle and topic (A “is” B and B “is” A; “death is a fat fly” and “a fat fly is death”) leads to the disclosure of emergent meanings [93]. Emergent meanings are attributes of the topic and vehicle that are not salient for either category considered separately but that become salient for both categories during metaphor comprehension (e.g., apropos death and fat flies, “lurking contamination”). There is now secure empirical evidence that emergent meanings derive from poetic metaphors [94,95,96,97], although it remains unclear how the integration of (A “is” B) and (B “is” A) generates emergent meanings.

To address the unfolding generation of oneiric emergent meanings, it is useful to examine the domain interaction model of metaphor comprehension [98,99]. The domain interaction model describes the juxtaposition of a topic and vehicle that are both “semantically dense” (concrete, embodied, self-relevant [100]) but that, at an abstract (hypernemic, taxonomic) level, are “distant” from each other. Using this model, it is possible to identify attributes of the metaphoric vehicle that are sufficiently abstract to also be attributes of the metaphoric topic and, in that way, support bidirectional metaphor comprehension. This model of metaphor comprehension suggests that the inclusiveness of an abstract (and ad hoc) ontological category may subsume two or more semantically dense regional ontological categories. The result is an abstract-ontological concept that is “inclusive” but not “totalizing”. Both existential and transcendent dreams generate that kind of metaphoric outcome.

#### 3.3.4. Metaphors of Personal Identification

A fourth important figurative relation is the self-relevant metaphoricity that provides a context for inexpressible realizations with a felt release. The iterative modulations that characterize the extended metaphors of existential and transcendent dreams (e.g., in the Hotel Dream) generate an unfolding sense of a meaning-finding self that Cohen [101] calls metaphors of personal identification. Consistent with Cohen’s account, there is fledgling evidence that engagement with nominal metaphors facilitates recognition of subsequent personifying metaphors [102] and that the cumulative effect of recurrent personifying metaphors is a sense of self as the author of unfolding metaphor extension and explication [103]. For example, in response to “all prayer is grief flying”, extended metaphoric modulation contributes to tacit endorsement of the following locutions: “I am [the ascent of] prayer”; “I am [the anguish of] grief”. The progressive integration of compound metaphors of personal identification—and their poignantly sublime conclusion during, for example, the Hotel Dream—contrasts with the unidirectional metaphoric representations that characterize retrospective attempts to identify waking concerns [37,38]. Moreover, the sublime aesthetic-epistemic effects of this progressive thematic integration during the dream differ from the individual personal insights derived from retrospective dream reflection [104].

## 4. Metacognition at the Abstract Ontological Limits of Sublime Feeling

Articulating a theory of oneiric movement toward metaphors of personal identification motivates comparison with Kant’s discussion of the radical limit experiences that constitute sublime feeling. Those radical limit experiences occur within a mode of metacognition that integrates *successive* metaphoric/literal metaphoric structures. Given that the tenets of Kant’s account have been altered by scholars working in the continental tradition [105], the present framework is best considered neo-Kantian.

Kant [106] proposed that interplay between a succession of sensible “aesthetic ideas” and insensible “rational concepts” moves toward “symbolic” (metaphoric) approximation of an abstract-ontological concept. Husserl [107] similarly described how interplay between a succession of intuitable (sensible) past, present, and anticipated “moments” move toward approximation of an abstract-ontological type. However, Kant does and Husserl does not address the metaphoricity of evolving abstract-ontological types. Consequently, Kant does, but Husserl does not, address the radical limits of “totalizing” abstract-ontological types.

### 4.1. Affective Awakenings

It is useful to begin by considering Husserl’s relatively accessible account of abstract-ontological types. In his (late) genetic phenomenology, Husserl developed the notion of a preconceptual type, which is comparable to the Kantian notion of a schema [108]. Whereas Kant argued that a schematic a priori is independent of nearly all sensible intuition, Husserl, already in *Logical Investigations II* [109] (§27), argued for the articulation of an “ideational abstraction” (*ideierende Abstraktion*) through a fully intuitable “eidetic variation” (*eidetische Variation*). A preconceptual type is initially generated through a temporal succession of adumbrations that is synthesized to provide a singular, intuitively given categorial object (e.g., “this” is “a doll”). In turn, articulation of an initial intuitively given categorial object enables an active explication of another intuitively given categorial object. One possible outcome is that the adumbrations originally gathered into a sensible intuition of the preconceptual type are “run through” again during confirming or disconfirming explication of the original type (“this” is [or is not] “another doll”). A second possibility is that the adumbrations may “give” another categorical object that differs from a previous determination, motivating alteration of the original preconceptual type (“this” is “not a doll” but rather “a mannequin”). A third possibility is that the adumbrations may not just alter but expand the original preconceptual type (“this” is a preconceptual type that subsumes both “mannequins and dolls”).

Husserl [109] (§27) gave special consideration to affectively “awakened” associations in which preconceptual types are in conflict. During a momentary “awakening”, he argued, two perceptual types “permeate” each other. Their permeation is apprehended as conflict, although both objects share a tenor of appearance (*Erscheinungsgehalt*). For Husserl, the tension in this permeation is resolved in a literal judgment (this is not a doll but rather a mannequin). He does not address the possibility that this permeation persists as a modifier-modified compound that provides adumbration of a relatively abstract ontological type that includes both doll-like mannequins and mannequin-like dolls.

### 4.2. Symbolic Hypotyposis

Both Kant and Husserl were alert to the ontology-expanding import of successions of associated categorial concepts (Kant as symbolic approximations of insensible *a priori* concepts; Husserl as the extended explication of permeating preconceptual types). In Kant’s account, orienting attunement toward a succession of aesthetic ideas initiates the “spontaneous” movement of “aesthetic ideas” toward “totalizing” concepts [110]. Kant argues that it does so by initiating a form of metaphoricity through which aesthetic ideas “approximate” rational conceptions of the “world as a whole”. He argues that the movement (*Schwung*) of the productive imagination toward totalizing concepts entails “symbolic” presentations that are “in accordance with an analogy” [106] (CPJ 5: 352). Such “symbolic hypotyposis”, he says, is pivotal when analogy offers the only way to exhibit (or present) rational concepts. Because Kant’s theory of symbolic hypotyposis resembles Aristotle’s theory of metaphor, it is possible to construe his account as a general theory of metaphor. However, Pillow [111] more specifically proposed that Kant’s theory of symbols resembles the “interactionist” theories of poetic metaphor [6,90,91,92]. Clarification of this theoretical possibility follows a path leading from Kant’s discussion of symbolic hypotyposis to Ricoeur’s discussion of “living metaphor”. Toward the end of this theoretical path, Ricoeur [7] presented living metaphor

“… as a mode of discourse that functions at the intersection of two domains, metaphorical and speculative. It is therefore a composite discourse, and as such cannot but feel the opposite pull of two rival demands. On one side, interpretation seeks the clarity of the [abstract ontological] concept; on the other, it hopes to preserve the dynamism of meaning that the [abstract ontological] concept holds and pins down. This is the situation Kant considers in the celebrated paragraph 49 of the Critique of the Faculty of Judgment. … [The interplay] in which imagination and understanding engage assumes a task assigned by the Ideas of reason, to which no [abstract ontological] concept is equal. But where the understanding fails, imagination still has the power of ‘presenting’ the Idea (*Darstellung*). It is this ‘presentation’ of the Idea by the imagination that forces conceptual thought to think more. Creative imagination is nothing other than this demand put to [abstract ontological] conceptual thought.”

(p. 303)

Ricoeur goes on to describe (a) how poetic metaphoricity entails “discourse [that is] turned back upon itself and spurred on by [another] metaphorical utterance”; (b) how such discourse is iteratively extended (perhaps indefinitely) to constitute a poetic theme; and (c) how poetic thematization approximates (without attaining) abstract ontological determination. Such iteratively extended and unfolding thematization does not simply reduce poesy to the explicative identification of abstract ontological concepts. Rather, such thematization moves toward further metaphoric modulation that persistently—but always inadequately—is sensed as an approximation of abstract ontological concepts.

### 4.3. Implications

Although it is difficult to investigate the chained images that give epistemic import to the themes emerging from extended metaphors, doing so is pivotal in studies of the unfolding metaphoricity of a literary text [112,113], the unfolding metaphoricity of the Borges lines, or the unfolding metaphoricity of the Hotel Dream. In the Borges excerpt, constitution of a relatively abstract (ad hoc) ontological type occurs as the permeations through which “time” is successively apprehended as a moving river, a devouring tiger, and an immolating fire (and then the integrative sense of this succession). These permeations are not literally “the same” but, considered metaphorically, they are “the same”. Moreover, this succession of permeations may provide adumbrations of an abstract ontological type that subsumes a network of subordinate ontological types (a network that subsumes the regional ontology of linear time, the regional ontology of moving rivers, the regional ontology of devouring tigers, and the regional ontology of immolating fires).

Similarly, constitution of a relatively abstract (ad hoc) ontological type may occur as the permeations through which “coercive intimacy” is successively apprehended as “worry about rapists”; as “unwelcome assistance to filial fellow travelers”; and as “aversion to a series of demanding voices” (and then as the integrative sense of this succession). Again, these permeations are not literally “the same” but, considered metaphorically, they are “the same”. Also, this succession of permeations potentially provides adumbrations of an abstract ontological type that subsumes a network of subordinate ontological types (a network that subsumes the regional ontology of coercive intimacy; the regional ontology of lurking rapists; the regional ontology of oblivious siblings; and the regional ontology of demanding voices).

## 5. Sublime Feeling: Disquietude and Enthrallment

The preceding framework begins to clarify the difference between (a) metacognition of the metaphoric/literal tension within local modifier-modified syntactic structures and (b) metacognition of the extending, expanding, and perhaps “totalizing” tensions that are the substrate of sublime feeling.

On one hand, within a local modifier-modified syntactic structure, a resolvable tension emerges between what metaphorically “is” and literally “is not”. While metaphor comprehension resists assimilative subsumption within familiar literal categories, it also invites accommodative subsumption within unfamiliar—and often ad hoc—semantically resonant categories. While an accommodation gradient extends from quasi-metaphoric resonance detection to unidirectional vehicle-topic mapping and the emergent meanings of bidirectional vehicle-topic mapping, an accommodating category expansion is usually possible.

On the other hand, successively extending, expanding, and perhaps “totalizing” metaphoric/literal syntactic structures may generate an irresolvable tension between abstract ontological thematization of what metaphorically “is” and literally “is not”. A radical epistemic limit may result from a succession of thematically coherent but conceptually inadequate quasi-metaphoric resonances, unidirectional vehicle-topic mappings, bidirectional metaphoric (emergent) meanings, and (eventually) “totalizing” abstract-ontological concepts. Within such “totalizing” metaphoric extension and expansion, accommodating category generation is often impossible [114], except as a further metaphoric extension of an abstract-ontological theme [7].

During existential and transcendent dreams, the dreamer may experience such extended and expanded but conceptually inadequate meanings as a cumulative (even “totalizing”) thematic tension between what metaphorically “is” and literally “is not”. However, whether such thematization moves toward further metaphoric modulation in “living metaphors” and whether further (“living”) metaphoric modulation is disquieting or enthralling depends upon dream type.

### 5.1. Sublime Disquietude

Recurrent and inevitably inadequate “symbolic” (metaphoric) approximations of “existential” (autobiographical) abstract-ontological concepts characterize oneiric sublime disquietude. That hypothesis is supported by studies [16,18] indicating that the carryover effects of existential dreams include disquieting doubt [115] (e.g., “… doubt became an important part of what it means to be honest about my existential concerns”). Also, the self-perceptual depth that consistently is reported following existential dreams [14,16,18,73] relies upon items that are not only self-relevant (e.g., “… I became sensitive to aspects of my life that I usually ignore”) but also relevant for humans in general (e.g., “… my sense of life seemed less superficial”). Consequently, the index of sublime disquietude used in ongoing research [116] incorporates the interactive combination of rated disquietude, self-perceptual depth, and inexpressible realizations. The carryover effects of existential dreams consistently involve sublime disquietude in this sense.

### 5.2. Sublime Enthrallment

In contrast, recurrent and inevitably inadequate “symbolic” (metaphoric) approximations of “transcendent” abstract-ontological concepts characterize sublime enthrallment. Sublime enthrallment arises through recurrent metaphoric/literal intimations of the possibilities and limits of ontological intersubjectivity. Intimations of ontological intersubjectivity (sensed as “awe”, “respect”) hover between (a) non-utilitarian (respectful) attunement to mutual intentionality within a dichotomous relationship [117] (*Ich und Du*) and (b) non-utilitarian (respectful) attunement to the “inner subjectivity” of beings in general (i.e., inclusively “totalizing” intersubjectivity).

The ontological intersubjectivity that characterizes sublime enthrallment is suggested by studies [16,18] indicating that the carryover effects of transcendent dreams include the apprehension of distributed liveliness (e.g., “… I had the sense that everything around me was somehow alive”). Also, the self-perceptual depth that is reported consistently after transcendent dreams [16,18] reflects not only poignant self-referential “depth” (e.g., “… I became sensitive to aspects of my life that I usually ignore”) but also the disclosure of “depth” in the experience of humans more generally (e.g., “… my sense of life seemed less superficial”). Consequently, the index of sublime enthrallment used in ongoing research [116] incorporates the interactive combination of rated reverence, self-perceptual depth, and inexpressible realizations. The carryover effects of transcendent dreams consistently involve sublime enthrallment in this sense.

In his portrayal of dialogical I-Thou relations, Buber emphasized the pivotal character of the “fresh running springs” of poetic language (cited in [118] p. 17), a locution that echoes Ricoeur’s account of “living metaphor” Although Stace [119] worried that the language of poetry was too “conceptual” to capture the ecstatic experience of the “inner subjectivity” of beings in general, psychometric studies have isolated a component of extravertive mystical experience (“distributed liveliness”) that is also evident in spiritual (secular) poetry [120] and in dreams [121,122]. Even so, these studies—and their authors—do not address the complexities of poetic metaphor, especially the extended and ontologically expanded themes that are disclosed in an unfolding succession of poetic metaphors.

#### Oneiric Sublime Enthrallment: An Example

At this point, it may be helpful to examine the metaphoric form of a prototypic transcendent dream [80] (p. 129–130):

***The Snake Dream.*** I dream that I am climbing a stairs, coming to a landing. There are windows on both sides [and] high walls. It’s very bright. Four people are there, and one person in particular is … interacting with me. He hands me a box that is gift-wrapped and … about shoe size. I start opening the box and then I [see it has] a cover [with] snakes [on it] … Obviously there is a feeling of friendship between us or a feeling of comfortable joking … and I ask, “Oh, you wouldn’t dare, would you?” And, he said, “Well, you’ll have to open it and find out”. // So I open the box and, when I open the box, this multitude, this large number of snakes come out of the box. They’re very thin, and they’re long like ribbons. They’re black, shiny, with yellow heads. They all wrap around my lower right leg like a cuff … I’m trying to push them away, to kick them off, feeling panicky, trying to … pry them off. And the more I try, the more I’m getting nowhere, the tighter they are wrapping themselves -- // until I suddenly calm down and start looking at them and see how they fit together and how they are not dangerous snakes. I start looking at them and I notice the color of their backs and how they seem to be so quiet and peaceful. So, I start touching them gently and, as I’m touching them, I’m talking to them. And, eventually I say, “OK, now you have to leave”, and they do leave… // When the snakes did leave, I felt like I was light, I was lighter in weight, but there was also a sense of release…

In this dream, successive episodes (separated here by //) provide semantically resonant representations through which the dreamer becomes more respectfully aware of the dangerous beauty of the snakes’ participation in distributed ontological intersubjectivity. In the first episode, the snakes’ presence is suggested by a pictorial image on the cover of a box that contains them; metaphorically their sentience is a teasingly hidden gift. In the second episode, the colorfully present snakes are disclosed in a threatening form that motivates the dreamer’s anxious resistance; metaphorically their sentience is antagonistic. In the third episode, the same colorfully presented snakes are quiet and peaceful, motivating the dreamer’s touch and dialogue; metaphorically their sentience is that of mutual intentionality. The snakes share the subjectivity of the dreamer; metaphorically their awareness—and the dreamer’s—is mutual and respectful. The culminating moment of “release” is not appreciation of a gift or relief from antagonistic threat—but rather non-utilitarian regard. The snakes retain their beauty (as in the decorated box); they retain their danger (they still “must leave”); but they also have the capacity for dialogue. Like the Hotel Dream, each successive metaphor deepens and enlivens without erasing its predecessor.

## 6. Conclusions

The results of the preceding analysis indicate that existential dreams transition into sublime disquietude and transcendent dreams transition into sublime enthrallment (while nightmares transition into post-awakening vigilance). This pattern prompts a reconsideration of when, if at all, nightmares generate poetic metaphors—and their aesthetic-epistemic aftereffects (contra Barcaro [4] and Hartmann & Kunzendorf [123]). Instead, this pattern is consistent with the notion that the transition into sublime feeling is precipitated by a distinctive form of metacognition specifically within existential and transcendent dreams. The two levels of metacognition within these two dream types may enable attunement to tension between the unfolding metaphoric and literal truth value of resonant categorial dream objects. The felt sense of that tension in these two dream types may disclose the unfolding epistemic—and aesthetic—significance of successive resonant images, culminating in the depth of sublime feeling.

*REM Sleep Neurophysiology*. The present account identifies oneiric sublime feeling as a distinctive REM sleep carryover effect. Examining the neurophysiology of that effect is a challenge because the infrequent and aperiodic character of impactful dreams effectively precludes their observation within a sleep laboratory. A comparable methodological limitation prevails in studies of sleep paralysis, although a few laboratory case studies indicate that isolated sleep paralysis episodes occupy an intermediate position between the cortical and neuromuscular activation of wakefulness and the cortical activation and muscle atonia of REM sleep. The result is that the dreamer is “partially aware” of the sleep environment [124], p. 723. Similarly, we suggest, during existential and transcendent dreams, the dreamer is partially aware of dream images that seem metaphorically real (present) but literally not real (not present).

There may be another reason to compare impactful dreams and sleep paralysis episodes. Cheyne and colleagues [125,126] reported three types of sleep paralysis episodes: (a) intruder episodes (multi-sensory hallucinations of a threatening figure); (b) incubus episodes (multi-sensory hallucinations of chest pressure, suffocation, and pain); and vestibular-motor episodes (illusory movement and out-of-body experiences). This tripartite typology is analogous to the tripartite typology of impactful dreams. Perhaps, then, existential dreams, transcendent dreams, and nightmares are muted or modified forms of the same tripartite psychobiological network that Cheyne et al. argue is the substrate of intruder, incubus, and vestibular-motor sleep paralysis episodes. If so, it would be useful to compare not only the underlying psychobiological networks of sleep paralysis episodes and impactful dreams but also the nuances of metacognitive epistemic attunements that accompany these exceptional oneiric moments.

Such comparisons—and any further examination of the hypotheses examined here—calls for psychometric ingenuity. One option is to use recently developed quantitative procedures to assess the semantic density (concreteness, embodiment, self-relevance) of basic level dream categories [100]. The complementary requirement is the development of systematic methods for assessing the semantic distance between dream categories and their relative level of abstraction [99,100]. Using enhanced computational procedures with category pairs identified as resonant by the dreamer may enable the prediction of metaphoric bidirectionality and emergent meanings [93].

Comparisons and hypothesis testing in this domain also call for a balance between psychometric rigor and connoisseurship. It will be important to attempt further articulation of the content categories that differentiate the metacognitive appraisals, inexpressible realizations, and moments of sublime feeling that are grounded in “living” aesthetic-epistemic metaphors. This may not be the place to expect operational definitions alone but rather the kind of connoisseurship that provides reliable articulation of the metaphoric structure of the Borges lines, existential dreams (e.g., the Hotel Dream), and transcendent dreams (e.g., the Snake Dream).

## Figures and Tables

**Table 1:** The contrasting features of nightmares, existential dreams, and transcendent dreams *.

	Nightmares	Existential Dreams	Transcendent Dreams
**Feelings** **and Emotions**	Fear/anxiety	Sadness/despair	Ecstasy/awe
**Narrative** **Themes**	Harm avoidance	Separation/loss	(Magical) goal attainment
**Movement** **Characteristics**	Energetic (evasive) movement	Movement inhibition (fatigue)	Graceful movement (floating)
**Sensory** **Anomalies**	---	Unusual light/dark contrasts	Extraordinary sources of light
**Spontaneous** **Transformations**	Physical metamorphoses	Spontaneous feeling shifts	Spontaneous perspective shifts
**Concluding** **Affective Tone**	Intense dream endings	Intense dream endings	Intense dream endings
**Metacognitive Stance**	---	Dual Perspectives	Dual Perspectives

* Table entries capture the gist of (a) two phenomenological studies involving a combination of first-person rating scales and open-ended dream description [14,15] and (b) three studies involving rating scales for the features identified in the original phenomenological studies [16,17,18].
